# Comparing recovery community centers (RCCs) serving Black, Hispanic/Latino, and other communities: an exploratory secondary data analysis of a nationwide survey of RCC directors

**DOI:** 10.3389/fpubh.2025.1532488

**Published:** 2025-07-02

**Authors:** Diadora DeCristofaro, Allison Futter, Alivia Williamson, Susanne S. Hoeppner, Lauren A. Hoffman, Marion J. Riggs, Judeline Joseph, Julia Ojeda, Amy A. Mericle, Vinod Rao, Philip X. Rutherford, Patty McCarthy, Bettina B. Hoeppner

**Affiliations:** ^1^Psychiatry Department, Massachusetts General Hospital, Boston, MA, United States; ^2^Department of Neurology, Harvard Medical School, Boston, MA, United States; ^3^Recovery Inspired Opportunities, Boston, MA, United States; ^4^Alcohol Research Group/Public Health Institute, Emeryville, CA, United States; ^5^Black Faces Black Voices, Rochester, MN, United States; ^6^Faces & Voices of Recovery, Washington, DC, United States

**Keywords:** recovery, recovery community centers, treatment models, disparities (health racial), community recovery capital

## Abstract

**Objective:**

Racial and ethnic disparities exist in opioid-related overdose death rates and engagement with substance use disorder (SUD) treatment. Emerging peer recovery support services (PRSS) show promise in engaging and supporting marginalized populations. Recovery community centers (RCCs) are an important and growing source of community-based PRSS. Our goal was to examine if RCCs serving Black, Hispanic/Latino, or other racial/ethnic communities successfully engage marginalized populations in their community and if there are differences in the service models and functioning of RCCs serving different racial/ethnic communities.

**Methods:**

We conducted exploratory secondary analyses of a nationwide survey of RCC directors (*n* = 122), in which directors described their RCC in terms of logistics, footprints, service model, linkages, services, and attitudes toward medication treatment. Analysis of variance and chi-square tests were used to compare RCCs serving different communities (i.e., Black, Hispanic/Latino, Other) on these variables, where “serving a Black/Hispanic/Latino community” was operationally defined as being in a ZIP code with more than double the national prevalence of Black (13.6%) and Hispanic/Latino (19.1%) individuals in the United States.

**Results:**

On average, the median [IQR] percentage of Black participants within RCCs serving Black communities was 45% [30–63%] (51% of residents in the RCCs' ZIP codes were Black); in RCCs serving Hispanic/Latino communities, 50% [28–60%] of RCC participants were Hispanic/Latino (57% of residents in the RCCs' ZIP codes were Hispanic/Latino). Across 70 variables describing the RCCs' service model and functioning, only two statistically significant differences emerged between RCCs serving Black, Hispanic/Latino, and other communities, using an alpha of 0.05. RCCs differed in offering 12-step mutual aid groups (lowest in RCCs serving Hispanic/Latino communities; *p* = 0.03) and the existence of direct collaboration with clinical sites providing medications for opioid use disorder (MOUD, most common in RCCs serving Black communities; *p* = 0.03).

**Conclusion:**

The overall RCC model appears to be consistent across racial/ethnic settings in terms of footprints, model of care, services offered, connection to relevant systems and organizations, and attitudes toward medications. Given the commonly observed racial/ethnic disparities in SUD care, the robustness of the RCC model across communities is promising.

## Introduction

Racially minoritized communities have been disproportionately affected by the opioid epidemic. Although there were more total overall deaths among non-Hispanic/Latino Whites relative to all other ethnic groups between 1999 and 2017, there was a more drastic rise in opioid-related overdose deaths among Hispanic/Latino and non-Hispanic/Latino Black individuals. Age-adjusted opioid-related death rates increased from 3.5 overdoses per 100,000 people (Hispanic/Latino and Black individuals) in 1999 to 6.8 (Hispanic/Latino individuals) and 12.9 (Black individuals) per 100,000 in 2017 (1). These disparities were amplified during the COVID-19 pandemic. In 2020, the overdose death rate among Black individuals surpassed that of White individuals for the first time since 1999, with Black individuals estimated to have a 16.3% higher overdose death rate (2). While opioids remain a leading driver of substance use-related mortality, research over the past years highlights a disproportionate rise in alcohol (3), amphetamine, cannabis (4), and cocaine-related deaths (5) among Black and Hispanic individuals compared to White individuals.

The U.S. Department of Health and Human Services (HHS) has acknowledged the need to advance racial equity in access to health care, treatment receipt within the justice system, and drug policy for underserved populations (6). Unfortunately, ongoing racial/ethnic disparities exist for treatment. Substance use disorder (SUD) treatment settings are subject to systemic racial barriers (e.g., stigma, distrust), which have hindered them in effectively engaging and retaining non-Hispanic/Latino Black populations, resulting in lower rates of treatment utilization, treatment completion, and equitable medication access to medications for opioid use disorder (MOUDs; e.g., buprenorphine, methadone) (7–13). Structural inequities, rooted in historical discrimination and marginalization, are intensified by social stigma, racial biases, and government-driven punitive measures against people who use substances such as opioids, alcohol, cocaine, methamphetamine, heroin, and cannabis. These barriers have disproportionately affected communities of color (12, 14, 15). Existing literature highlights the impact of discrimination-induced psychological stress and its linkage with problematic substance use (12, 16). Structural and societal conditions have continued to perpetuate disparities in the quality of and access to healthcare, education, economic stability, and community resources (e.g., food, shelter, and medical needs). Indeed, poor socioeconomic conditions have been highlighted as contributors to lower treatment completion rates in Black and Hispanic/Latino populations compared to White populations in formal treatment settings (17–19). These factors, referred to as social determinants of health, are crucial to consider when highlighting the disproportionate rates of substance-use mortality for ethnic and racially minoritized populations (20–22).

The concept of recovery has increasingly shifted toward a more inclusive definition that recognizes its multifaceted nature. The Substance Abuse and Mental Health Services Administration's (SAMHSA) working definition describes recovery as “a process of change through which individuals improve their health and wellness, live a self-directed life, and strive to reach their full potential”. This definition emphasizes four key dimensions that support long-term recovery: health, home, purpose, and community (23). Recovery from SUD may not be achieved through clinical treatment for SUD alone; people follow diverse trajectories from SUD to recovery or remission, including through the help of mutual aid groups, treatment, and the provision of recovery support services (19). The need for recovery support services as part of clinical treatment or as a stand-alone path to recovery initiation and maintenance has given rise to peer recovery support services (PRSS), which are peer-driven non-clinical services delivered by trained peers, often called “peer recovery specialists” or “recovery coaches”. Although little research has been conducted to date, available studies suggest that PRSS have the potential to improve engagement in clinical treatment, as well as in other recovery-oriented settings (e.g., recovery community centers, transitional outpatient, and residential treatment centers) (24). A recent systematic review found that PRSS in hospital settings was found to facilitate linkages to treatment and follow-up care/support (24). Additionally, a positive relationship has been observed between PRSS participation and MOUD initiation, increased treatment retention, and greater treatment satisfaction. These studies point to the potential importance of PRSS in the recovery process, yet further research is needed, particularly regarding its delivery within the context of racial and ethnic disparities in SUD treatment and recovery.

One type of PRSS that may be particularly promising in addressing health disparities is recovery community centers (RCCs). RCCs are brick and mortar buildings that provide a “one-stop-shop” for recovery support services (25). Located within their communities, RCCs welcome all pathways of recovery and uphold and promote safe spaces for individuals regardless of the services they choose to engage with (25, 26). They provide a variety of services, including recovery coaching, support group meetings, relapse prevention skills-building, opioid and/or harm reduction services, technology and internet access, employment and job training linkages, assistance with basic needs and social services, substance-free recreational activities, health and nutrition programs, and civic participation (27). Beyond substance use disorders, RCCs also address behavioral and process addictions through SMART Recovery meetings, a mutual-help program grounded in cognitive behavioral therapy that explicitly supports recovery from behavioral addictions such as gambling, overeating, or internet use, thus supporting individuals across diverse recovery pathways (28). A nationwide survey of RCCs (*n* = 122) found that all offered support for alcohol and drug-related issues, while nearly 70% of RCCs also addressed “other addictions” and mental health concerns (29). Additionally, an online survey of RCC participants indicated that over 80% of RCC members engaged in polysubstance use, with opioids (32.7%) and alcohol (26.8%) reported as the most common primary substance (25).

RCCs provide extensive internal and external resources that can help individuals initiate and sustain recovery, and are an essential component in improving quality of life outside of clinical care (30). RCCs provide recovery support services that are especially useful to those in low-income communities who experience disparities in access to formal SUD treatment (29). Thus, RCCs may offer a more accessible and engaging support system for racially minoritized individuals by addressing key social determinants of health. Unlike traditional clinical settings, which primarily focus on SUD treatment, RCCs seemingly adopt a holistic approach—connecting individuals to essential social determinants of health that directly impact long-term recovery and stability. By integrating these broader support services, RCCs recognize that sustained recovery is not solely a medical issue (26, 27) but is deeply tied to structural inequities pertaining to socioeconomic health that disproportionately affect racially minoritized communities.

Importantly, RCCs appear to be able to engage ethnic and racially minoritized groups (29). This sets them apart not only from formal treatment settings, as described above but also from some of the more traditional PRSS. For example, recent membership surveys of Alcohol Anonymous (AA) and Narcotics Anonymous (NA) have shown relatively low engagement of Black and Hispanic/Latino people (3–13%) (31, 32). RCCs, on the other hand, may provide a welcoming environment to address disparities and increase recovery resources among Black and Hispanic/Latino populations (29). In this secondary data analysis of a nationwide survey of RCCs (29), we examine if the RCC service model shows a pattern of ethnic/racial disparities that is frequently observed in other SUD and opioid use disorder treatment and recovery settings. In particular, we utilize existing data to test if differences exist between RCCs serving Black, Hispanic/Latino, and other communities in terms of their reach, organizational capacity, service model, interconnectivity with other SUD-relevant organizations, and their attitudes toward MOUDs.

## Methods

### Sample

This is a secondary data analysis of a sample of RCC directors or delegates who participated in a nationwide survey of RCCs known at the time of the survey (29). Of the 198 RCCs contacted, 122 RCCs responded and completed the survey (122/198, 62% response rate) (29). We asked the directors of RCCs or their delegates to report on the logistics, footprint, model of care, demographics of RCC attendees, services provided, and linkages established at their RCCs (29). For the study, RCCs were defined as: “Brick and mortar places located within the heart of a community that serves as a central recovery hub by providing a variety of support services for people in or seeking recovery” (29).

### Procedure

For this secondary data analysis, we differentiated three different kinds of RCCs: RCCs serving Black communities, Hispanic/Latino communities, and other communities. To make these determinations, we utilized the publicly available physical address data for all RCCs in conjunction with 2021 U.S. Census ZIP code tabulation data on race and ethnicity (33). Our operational definition for RCCs serving a Black or Hispanic/Latino community was any RCC located in a ZIP code with more than double the U.S. national prevalence of Black (13.6%) or Hispanic/Latino (19.1%) individuals, respectively (33). Specifically, we defined an RCC as serving a Black community if it was located in a ZIP code in which more than 27.2% of the residents reported being Black, as per the U.S. Census; we defined an RCC as serving a Hispanic/Latino community if more than 38.2% of the residents reported being Hispanic/Latino, as per the U.S. Census. For RCCs with both high Black and Hispanic/Latino prevalence (*n* = 4), we grouped them according to their higher percentage. We selected this cutoff to capture ZIP codes with a substantial proportion of Black and Hispanic individuals. The 'other' category includes RCCs serving predominantly White populations as well as those serving racially/ethnically mixed or non-specified populations. Using a threshold set at twice the national percentage provides a more meaningful criterion for identifying RCCs that are more closely representative of and inclusive of these racial and ethnic groups.

Study data were collected and managed using the Research Electronic Data Capture (REDCap) system, hosted at Mass General Brigham. REDCap is a secure, web-based, and HIPAA-compliant software platform designed to support data capture for research studies (34). Study staff sent email invitations to the directors of each RCC and followed up via phone calls on three or more different days to remind participants to complete surveys, as needed. RCC leadership was offered $50 to complete the survey; 41 (34%) opted out of remuneration. The Mass General Brigham Institutional Review Board reviewed and approved all study procedures.

### Measures

RCC logistics, footprints, model of care, and demographics (Table 1) were assessed with multiple choice items and open-ended questions. To describe the demographics of the people served by their RCC, RCC directors or delegates were asked to estimate the percentage of RCC members per given category for age, gender, race, and ethnicity, as shown in Table 1. For example, when reporting on the age of their RCC members, RCC directors were asked to estimate the percentage of RCC members who were aged “under 18 years”, “18–24 years”, “25–59 years”, and “60+ years”. If percentages did not add up to 100%, the survey platform alerted them and asked for a correction (34). These estimates are based on reports from RCC delegates rather than intake forms or verified data, as many RCCs do not require formal sign-up or intake processes for individuals accessing walk-in services in these community-based settings.

**Table 1:** RCC logistics, footprints, model of care, and demographics across RCCs that serve different communities.

**Survey Questions**	**Total**		**Black**		**Hispanic/Latino**		**Other**		**Group difference**
	***n*** = **122**		***n*** = **23**		***n*** = **18**		***n*** = **81**		
	**M/Md/%**	**(SD/IQR/** ***n*** **)**	**M/Md/%**	**(SD/IQR/** ***n*** **)**	**M/Md/%**	**(SD/IQR/** ***n*** **)**	**M/Md/%**	**(SD/IQR/** ***n*** **)**	***p***
**RCC logistics**									
Number of years in operation, Md [IQR]^a^	5.00	[3, 10]	6.0	[4, 17]	7.0	[2, 11]	4.8	[2.5, 9.5]	0.02
Number of paid staff, Md [IQR]^a^	5.0	[3, 10]	5.0	[4, 10]	4.0	[2, 6]	5.0	[3, 10]	0.23
Number of volunteer staff at your RCC, Md [IQR]^a^	5.0	[3, 12]	7.0	[3, 14]	7.5	[3, 20]	5.0	[4, 10]	0.89
**Provides support for (*****% of RCCs*****)**, ***M*** **(SD)**									
Alcohol problems	100.0	(122)	100.0	(23)	100.0	(18)	100.0	(81)	
Drug problems	100.0	(122)	100.0	(23)	100.0	(18)	100.0	(81)	
Other addictions	69.7	(85)	60.9	(14)	72.2	(13)	71.6	(58)	0.59
Mental health problems	67.2	(82)	78.3	(18)	55.6	(10)	66.7	(54)	0.30
**RCC Footprints (in medians [IQR], due to skew)** ^a^									
Number of RCC members last year	500	[250, 2,150]	750	[250, 2,822]	450	[200, 1,600]	500	[290, 2,150]	0.63
Number of active RCC members last month	125	[50, 300]	150	[50, 350]	133	[30, 300]	110	[50, 300]	0.68
**RCC model of care, % yes (** ***n*** **)**									
A social place where people go to meet and spend time with peers (%/*n*)	77.0	(94)	69.6	(16)	83.3	(15)	77.8	(63)	0.56
A service-oriented place where people use services hosted by the RCC (%/*n*)	87.7	(107)	95.7	(22)	83.3	(15)	86.4	(70)	0.42
An information-oriented place where people are connected with resources and learn more about recovery (%/*n*)	91.0	(111)	87.0	(20)	94.4	(17)	91.4	(74)	0.72
**RCC demographics (** ***estimated by RCC director*** **)**									
Age (*% of RCC members in each age group*)									
< 25 years, M(SD)^b^	23.5	(17.1)	21.5	(12.1)	18.3	(14.0)	25.4	(18.7)	0.24
25–59 years, *M* (SD)^b^	63.3	(17.7)	63.7	(16.8)	61.0	(16.1)	63.7	(18.5)	0.84
60+ years, Md [IQR]^a^	10.0	[5, 17]	10.0	[5, 19]	16.5	[10,20]	10.0	[5, 15]	0.1428
Gender (*% of RCC members in each group*)									
Female, *M* (SD)^b^	42.7	(12.7)	44.2	(16.2)	37.5	(11.4)	43.5	(11.6)	0.18
Male, *M* (SD)^b^	55.3	(12.9)	52.6	(17.3)	60.4	(11.4)	54.9	(11.5)	0.16
Other, Md [IQR]^c^	0.0	[0, 2]	1.0	[0, 4]	1.0	[0, 5]	0.0	[0, 2]	all *p* > 0.25
Race (*% of RCC members in each group*)									
American Indian or Alaskan Native, Md [IQR]^c^	1.0	[0, 2]	1.0	[0, 1]	1.0	[0, 3]	1.0	[0, 2]	all *p* > 0.25
Asian, Md [IQR]^c^	1.0	[0, 2]	1.0	[0, 2]	1.0	[0, 5]	1.0	[0, 2]	n/a
Black/African American, Md [IQR]^a^	15.0	[6, 30]	45.0	[30, 63]	20.0	[13, 36]	10.0	[5, 20]	< 0.0001
Native Hawaiian or Pacific Islander, Md [IQR]^c^	0.0	[0, 1]	0.0	[0, 1]	0.0	[0, 2.5]	0.0	[0, 1]	n/a
White, *M* (SD)^b^	62.8	(24.2)	38.7	(17.5)	49.6	(26.0)	73.3	(18.1)	< 0.0001
More than one race, Md [IQR]^a^	8.0	[3, 15]	11.0	[4, 20]	10.0	[0, 17]	6.0	[3, 13]	0.94
Ethnicity (% Hispanic), Md [IQR]^a^	15.0	[5, 30]	15.0	[10, 30]	50.0	[28, 60]	10.0	[5, 20]	< 0.0001

RCCs serving other communities were all remaining RCCs that were not identified as serving either Black or Hispanic communities.

Wilcoxon rank-sum tests were performed on variables where assumptions of homogeneity and normality were violated. For RCCs who identified as Asian, there was a significant difference (*p* < 0.25) between RCCs serving Other communities and RCCs serving Hispanic/Latino communities (*p* = 0.0957). For RCCs who identified as Native Hawaiian or Pacific Islander, there was a significant difference (*p* < 0.25) between RCCs serving Other communities and RCCs serving Hispanic/Latino communities (*p* = 0.0957).

a Log-transformed data ANOVAs.

b Raw data ANOVAs.

c Wilcoxon rank-sum tests.

For RCC services (Table 2), RCC directors or delegates used a checklist (yes/no) to indicate the services their RCC provided. This checklist was derived from earlier research on RCCs located in the northeast of the U.S. (25, 35, 36). To describe the degree to which RCCs interacted with other organizations, they were asked: “Does your RCC currently have linkages to any of the following?”, and then were provided with checkboxes for a variety of types of organizations (as shown in Table 3).

**Table 2:** RCC services provided by RCCs serving different communities.

**Survey Questions**	**Total**		**Black**		**Hispanic/Latino**		**Other**		**Group difference**
	***n*** = **122**		***n*** = **23**		***n*** = **18**		***n*** = **81**		
	**%**	**(** ***n*** **)**	**%**	**(** ***n*** **)**	**%**	**(** ***n*** **)**	**%**	**(** ***n*** **)**	***p***
**Support group meetings**									
“All Recovery” meetings	72.1	(88)	78.3	(18)	61.1	(11)	72.8	(59)	0.46
Peer-facilitated recovery support groups (e.g., relapse prevention groups)	89.3	(109)	95.7	(22)	77.8	(14)	90.1	(73)	0.20
Mutual-help groups (e.g., Alcoholics Anonymous)	70.5	(86)	78.3	(18)	44.4	(8)	74.1	(60)	0.0296
Mental health support (e.g., dual diagnosis support groups)	54.1	(66)	65.2	(15)	50.0	(9)	51.9	(42)	0.49
**Recovery coaching**	82.8	(101)	91.3	(21)	77.8	(14)	81.5	(66)	0.52
**Opioid and/or harm reduction services**									
Medication-assisted treatment (MAT) support (e.g., Pathway Guide, MARS group)	42.6	(52)	52.2	(12)	38.9	(7)	40.7	(33)	0.58
NARCAN training and/or distribution	84.4	(103)	87.0	(20)	72.2	(13)	86.4	(70)	0.32
**Technology/internet access (e.g., use of center computers, printers, fax)**	73.0	(89)	73.9	(17)	72.2	(13)	72.8	(59)	0.99
**Assistance with basic needs and social services**									
Employment assistance (e.g., job or computer skills, resume writing, CORI support)	72.1	(88)	87.0	(20)	66.7	(12)	69.1	(56)	0.21
Basic needs assistance (e.g., access to food, clothing, transportation)	72.1	(88)	91.3	(21)	72.2	(13)	66.7	(54)	0.07
Family support services (e.g., family/parent education or support groups)	66.4	(81)	60.9	(14)	66.7	(12)	67.9	(55)	0.82
Housing assistance	63.9	(78)	69.6	(16)	50.0	(9)	65.4	(53)	0.38
Education assistance	53.3	(65)	47.8	(11)	44.4	(8)	56.8	(46)	0.54
Financial services	36.9	(45)	56.5	(13)	33.3	(6)	32.1	(26)	0.10
Health insurance education	36.1	(44)	47.8	(11)	22.2	(4)	35.8	(29)	0.24
Legal assistance	24.6	(30)	26.1	(6)	27.8	(5)	23.5	(19)	0.91
Childcare services	0.1	(10)	0.0	(0)	16.7	(3)	8.6	(7)	0.13
**Assistance with health behaviors**									
Health, exercise, and nutrition programs (e.g., fitness classes)	61.5	(75)	65.2	(15)	55.6	(10)	61.7	(50)	0.82
Smoking cessation support	17.2	(21)	17.4	(4)	22.2	(4)	16.1	(13)	0.77
**Facilitation of substance-free recreational activities**									
Recreational/social activities (e.g., substance free social events)	82.0	(100)	78.3	(18)	72.2	(13)	85.2	(69)	0.34
Expressive arts (e.g., arts/craft groups, music, poetry)	65.6	(80)	65.2	(15)	72.2	(13)	64.2	(52)	0.81
**Opportunity to volunteer/“give back” to the center**	89.3	(109)	95.7	(22)	83.3	(15)	88.9	(72)	0.42
**Recovery advocacy outreach and opportunities (e.g., community, regional, statewide events)**	84.4	(103)	82.6	(19)	77.8	(14)	86.4	(70)	0.57

**Table 3:** Organizations and systems RCCs connect with in fulfilling their mission across RCCs serving different communities.

**Survey Questions**	**Total**		**Black**		**Hispanic/Latino**		**Other**		**Group difference**
	***n*** = **122**		***n*** = **23**		***n*** = **18**		***n*** = **81**		
	***M*** **/%**	**(SD/** ***n*** **)**	***M*** **/%**	**(SD/** ***n*** **)**	***M*** **/%**	**(SD/** ***n*** **)**	***M*** **/%**	**(SD/** ***n*** **)**	***p***
**Does your RCC currently have linkages to any of the following?**									
Medical centers	80.3	(98)	87.0	(20)	77.8	(14)	79.0	(64)	0.79
Substance use disorder clinics	88.5	(108)	95.7	(22)	88.9	(16)	86.4	(70)	0.53
Clinics/prescribers who prescribe medication for substance use disorder	86.1	(105)	95.7	(22)	88.9	(16)	82.7	(67)	0.28
Behavioral treatment (individual/group therapy)	84.4	(103)	91.3	(21)	88.9	(16)	81.5	(66)	0.53
Emergency departments	72.1	(88)	69.6	(16)	66.7	(12)	74.1	(60)	0.76
Churches or other religious centers	72.1	(88)	78.3	(18)	72.2	(13)	70.4	(57)	0.76
Sober Homes	85.2	(104)	82.6	(19)	83.3	(15)	86.4	(70)	0.80
Criminal legal system (*originally called “justice system” in survey*)	78.7	(96)	78.3	(18)	77.8	(14)	79.0	(64)	1.00
Other non-RCC service/organization	31.1	(38)	39.1	(9)	22.2	(4)	30.9	(25)	0.51
**Do you wish that your RCC had more and/or better direct linkages to any of the following? Select ALL that apply?**									
Medical centers	33.6	(41)	30.4	(7)	50.0	(9)	30.9	(25)	0.28
Substance use disorder clinics	29.5	(36)	26.1	(6)	44.4	(8)	27.2	(22)	0.32
Clinics/prescribers who prescribe medication for substance use disorder	36.1	(44)	30.4	(7)	50.0	(9)	34.6	(28)	0.38
Behavioral treatment (individual/group therapy)	27.9	(34)	30.4	(7)	44.4	(8)	23.5	(19)	0.19
Emergency departments	33.6	(41)	43.5	(10)	22.2	(4)	33.3	(27)	0.36
Churches or other religious centers	18.9	(23)	26.1	(6)	27.8	(5)	14.8	(12)	0.24
Sober Homes	29.5	(36)	39.1	(9)	44.4	(8)	23.5	(19)	0.11
Justice system	24.6	(30)	21.7	(5)	27.8	(5)	24.7	(20)	0.90
Other non-RCC service/organization	10.7	(13)	13.0	(3)	16.7	(3)	8.6	(7)	0.51

RCC directors were also asked to provide feedback on their RCC's approach to SUD medication treatment in general (Table 4). We asked: “How open is your RCC to medication-assisted treatment?” with answer options rated on a 1–5 scale, ranging from 1 (“not open at all”) to 5 (“extremely open”). We then asked RCC directors and delegates to indicate in a checklist how they handled MOUDs specifically (“How does your RCC handle medications for opioid use disorder? Select ALL that apply”; response options are shown in Table 4).

**Table 4:** RCC attitudes toward medication assisted recovery across RCCs serving different communities.

**Survey Questions**	**Total**		**Black**		**Hispanic/Latino**		**Other**		**Group difference**
	***n*** = **122**		***n*** = **23**		***n*** = **18**		***n*** = **81**		
	***M*** **/Md/%**	**(SD/IQR/** ***n*** **)**	***M*** **/Md/%**	**(SD/IQR/** ***n*** **)**	***M*** **/Md/%**	**(SD/IQR/** ***n*** **)**	***M*** **/Md/%**	**(SD/IQR/** ***n*** **)**	***p***
**Openness of RCC to medication-assisted treatment (scale 1–5)**									
Average score (on 1–5 scale) Md [IQR]^a^	5.0	[4, 5]	5.0	[4, 5]	5.0	[4, 5]	5.0	[5, 5]	all *p* > 0.25
Percent of RCCs indicating “extremely open” (%/*n*)	68.0	(83)	66.7	(14)	72.2	(13)	75.7	(56)	0.40
**RCC's handling of medications for opioid use disorder (MOUDs)**									
Provides direct support for MOUDs (e.g., providing information) (%/*n*)	77.0	(94)	82.6	(19)	83.3	(15)	74.1	(60)	0.55
Staff engage members in conversations about MOUDs (%/*n*)	85.2	(104)	82.6	(19)	94.4	(17)	84.0	(68)	0.56
RCC works directly with clinical sites providing MOUDs (%/*n*)	63.9	(78)	87.0	(20)	50.0	(9)	60.5	(49)	0.0271
RCC does proactive outreach to persons using MOUDs (%/*n*)	57.4	(70)	65.2	(15)	55.6	(10)	55.6	(45)	0.70
RCC advocates that people use MOUDs (%/*n*)	45.9	(56)	52.2	(12)	50.0	(9)	43.2	(35)	0.70
RCC tolerates use of MOUDs, but does not actively encourage it (%/*n*)	1.6	(2)	0.0	(0)	0.0	(0)	2.5	(2)	1.00
RCC discourages people from starting MOUDs (%/*n*)	0.0	(0)	0.0	(0)	0.0	(0)	0.0	(0)	n/a
RCC advises people to stop using MOUDs (%/*n*)	0.0	(0)	0.0	(0)	0.0	(0)	0.0	(0)	n/a
Does not apply—RCC does not have members with opioid use disorder (%/*n*)	0.0	(0)	0.0	(0)	0.0	(0)	0.0	(0)	n/a

RCCs serving other communities were all remaining RCCs that were not identified as serving either Black or Hispanic communities.

Wilcoxon rank-sum tests were performed on variables where assumptions of homogeneity and normality were violated (all *p* > 0.25).

a Log-transformed data ANOVAs.

### Analytic strategy

To compare RCCs serving primarily Black (*n* = 23) vs. Hispanic/Latino (*n* = 18) vs. other communities (*n* = 81), we calculated descriptive statistics [i.e., means (*M*) with standard deviations (SD) for continuous variables; medians (Md) with interquartile ranges [IQR] for continuous variables with substantial skew; and percentages (%) with sample sizes (*n*) for categorical data (yes/no)] for survey items. For variables where RCC directors or their delegates estimated percentages for their RCC (i.e., RCC member percentages in different age, gender, race, and ethnicity categories), we treated the percentage estimates provided by RCCs as continuous variables. For continuous and count variables, we conducted group comparison tests via one-way analysis of variance (ANOVA); where necessary, we log-transformed data to better meet the assumptions of homogeneity of variance and normality (37, 38). For continuous variables with substantial skew even after log-transformation [e.g., “Other” genders, American Indian or Alaskan Native race, Asian race, and Native Hawaiian or Pacific Islander race, as well as for the variable *Openness of RCC to medication-assisted treatment* (scale 1–5)], we used non-parametric Wilcoxon rank-sum tests to test pairwise group differences (for details, see Supplementary material). For categorical variables, we used chi-square tests to compare RCCs serving primarily Black, Hispanic/Latino, or other communities. Due to the exploratory nature of these analyses, we did not adjust the group effect tests for multiple comparisons. For group difference tests that showed overall group differences at a significance level of *p* < 0.25 (a common threshold in screening for univariate predictors) (39), we conducted follow-up pairwise comparisons to further evaluate specific group differences (40, 41). We adjusted the pairwise group differences for multiple comparisons using the Holm-Bonferroni procedure at a significance level of α = 0.05 to detect differences (42).

## Results

### Engagement of Black and Hispanic/Latino individuals in RCC participation

To descriptively assess equity in RCC engagement, we compared the proportion of Black and Hispanic/Latino participants reported by RCCs to the racial/ethnic composition of the communities in which the RCCs are located. While not a causal analysis, this comparison offers preliminary insight into whether RCCs are reaching racially minoritized populations at or above expected levels based on local demographics. RCCs serving Black and Hispanic/Latino communities successfully engaged Black and Hispanic/Latino individuals in recovery support services. That is, of RCCs serving Black communities, the median [IQR] percentage of RCC participants who were Black was 45% [30–63%], compared to an average of 51% (SD = 21) of Black people living in the surrounding zip code. Similarly, in RCCs serving Hispanic/Latino communities, the percentage of RCC participants who were Hispanic was 50% [28–60%], compared to 56.8% (SD = 16.6) Hispanic/Latino people living in the surrounding zip code. Paired *t*-tests of these differences (i.e., RCC participant racial/ethnic prevalence vs. local zip code racial/ethnic prevalence) were not significant (*p* = 0.18) for RCCs serving Black communities, *p* = 0.06 for RCCs serving Hispanic/Latino communities).

### Differences in other demographics

A significant difference was found in the number of years RCCs had been in operation across the groups (*p* = 0.02, based on an ANOVA of log-transformed data). Median [IQR] years in operation were 6 (4–17) for RCCs serving Black communities, 7 (2–11) for RCCs serving Hispanic/Latino communities, and 4.8 [2.5–9.5] for RCCs serving other communities, respectively. Although no pairwise comparisons reached statistical significance after adjustment, the comparison between RCCs serving Black communities and those serving other communities approached significance and accounted for most of the observed group-level difference (*p* = 0.08).

### Differences in services and service model

Across 52 characteristics, including 23 possible services offered (Table 2), 18 possible linkages provided (Table 3), and 11 descriptors of how RCCs handled medication assisted recovery (Table 4), only two statistically significant overall group differences emerged between RCCs serving Black, Hispanic/Latino, and other communities. Of note, given an alpha of 0.05, 2–3 significant differences (i.e., 5%) would be expected to emerge by chance. Importantly, RCCs in this sample demonstrate an all-encompassing approach to supporting long-term recovery. A notable number of RCCs reported providing support for addictions apart from substance use (69.7%; Table 1) and mental health problems (67.2%; Table 1). Where the literature has demonstrated that co-morbidities often occur in SUD and other addictions, such as mental health problems (43), RCCs provide resources that address all aspects of recovery, suggesting a comprehensive approach to acknowledging and supporting the many facets and complexities of sustaining recovery.

The two differences observed were in the offering of 12-step groups (*p* = 0.0296; Table 2) and linkages to clinics that provide MOUDs (*p* = 0.0271; Table 4). Offering 12-step mutual aid groups (e.g., Alcoholics Anonymous) was least common in RCCs serving Hispanic/Latino communities (44.4%) and considerably more common among RCCs serving Black communities (78.3%; *p* = 0.05) and other communities (74.1%; *p* = 0.04); there was no difference in the prevalence of offering 12-step groups between RCCs serving Black or other communities (*p* = 0.69). In terms of linkage to medication, direct collaboration with clinical sites providing MOUDs was most common in RCCs serving Black communities (87.0%) compared to RCCs serving Hispanic/Latino communities (50.0%; *p* = 0.03) or other communities (60.5%; *p* = 0.04); there was no difference in the prevalence of direct collaboration with clinical sites providing MOUDs between RCCs serving Hispanic/Latino or other communities (*p* = 0.42).

### Exploratory *post-hoc* analysis

Exploratory Holm-Bonferroni adjusted *post-hoc* analysis, triggered by group difference tests with a *p* < 0.25, showed additional differences in aiding with basic needs (Table 2) and social services (Table 3). Namely, although non-significant, RCCs serving Black communities demonstrated a greater ability to provide basic needs assistance (e.g., access to food, clothing, transportation; 91.3% of RCCs) compared to RCCs serving other communities (66.7% of RCCs; *p* = 0.06) and were more likely to provide financial services (56.5% for RCCs serving Black communities) compared RCCs serving other communities (32.1%; *p* = 0.06).

Lastly, while we were unable to detect any differences in the prevalence of current linkages between RCCs and other organizations and systems that RCCs connect with in fulfilling their mission (all *p* > 0.25; Table 3), there were two differences in the prevalence of RCC directors or their delegates who wished they had more and/or better direct linkages. Across all options for linkages except emergency departments, directors and delegates of RCCs serving Hispanic/Latino communities more frequently expressed a desire for expanded and improved linkages. Particularly, RCCs serving Hispanic/Latino communities were more likely to express a wish for more and better linkages to behavioral treatment (i.e., individual/group therapy; 44.4%) and sober homes (44.4%) compared to RCCs serving other communities (behavioral treatment: 23.5%, *p* = 0.0718; sober homes: 23.5%, *p* = 0.0718), with RCCs serving Black communities at intermediate prevalences that showed no difference to either of the other types of communities (all *p* > 0.10).

## Discussion

In conducting this secondary data analysis examining RCCs in Black, Hispanic/Latino, and other communities, we found that while each RCC is unique, the overall service and support model in the RCCs included in this sample appears to be consistent across racial/ethnic community settings. This consistency includes their range of operation, model of care, services offered, connection and linkages to relevant systems and organizations, director openness, support, and engagement with participants utilizing MOUDs, regardless of demographic composition. RCCs have gained attention as a valuable recovery support service and community resource over the past two decades (44). Given that there are a limited number of RCCs, future research should further examine how RCCs serving specific racially minoritized individuals may be pivotal connections to lessen the gap between treatment and sustained recovery. RCCs operate in environments where racial disparities are commonly observed. However, within our sample, we do not see evidence of service and support disparities among RCCs serving Black, Hispanic/Latino, and other communities—an encouraging finding. Further research is needed to establish this.

We noted two specific differences in linkages between RCCs and other systems and organizations that support or work with people seeking or in recovery. The first was that mutual aid organization linkage was significantly lower at RCCs serving Hispanic/Latino communities than at RCCs serving either Black or other communities. This reflects a national trend in which Black individuals attend mutual-help groups at slightly higher rates than Hispanic/Latino individuals (45–47). The more encouraging second difference was that direct collaborations between RCCs with clinical sites providing MOUDs were most common in RCCs serving Black communities (87.0% of RCCs). As previously noted, structural inequities and racial biases within SUD treatment systems have led to lower treatment engagement by Black individuals; however, our results show that RCCs serving Black communities may be actively seeking connections with MOUD clinics, which may be a pivotal step to reducing disparities in treatment seeking behaviors. Further research is needed to explore how connections between community-oriented supports, such as RCCs, and evidence-based treatment approaches, including mutual-help groups and MOUD clinics, may enhance recovery stability beyond traditional treatment settings.

Another set of interesting observations was that RCCs serving Black communities tended to provide basic needs assistance and financial services more frequently than other communities. While these results were not statistically significant, they may indicate that RCCs might be particularly responsive to the needs of their surrounding communities due to their reliance on community-based staffing, including PRSS, and their community-oriented treatment model. Given the disparities in health insurance coverage and financial resources among Black communities (16, 17), RCCs may be more inclined to prioritize financial assistance and access-related services to effectively address these barriers. The provision of such services can have meaningful impacts on the lives of recipients by helping them meet their basic needs and reducing barriers to their ongoing recovery. For instance, the provision of transportation assistance may enable recipients to attend recovery-related services and activities or hold jobs; the provision of clothing assistance may enable recipients to start and keep jobs and enhance their sense of dignity and self-worth; the provision of childcare services can help recipients regain or keep custody of their children, provide greater flexibility in attending recovery-related activities, and increase their ability to work and pay rent (48). Future research should examine if this increased service assistance was provided in response to greater economic need in the specific local communities they serve or whether this may indicate an unmet need in RCCs serving other communities. More work is needed to quantify the numerous benefits of these supportive services in helping individuals in recovery build recovery capital and enable more RCCs to offer these services.

Notably, 19–36% of all RCCs expressed a desire for more and better linkages to existing systems and organizations, most commonly to medical centers, clinics/prescribers who prescribe medication for substance use disorders, and emergency departments. RCCs serving Hispanic/Latino communities were, furthermore, particularly interested in more and better linkages to behavioral treatment providers and sober homes. More work is needed to strengthen and increase these existing linkages while ensuring that the organizations and systems that are already linked offer language-compatible and culturally appropriate services for Hispanic/Latino individuals seeking or in recovery. A study on substance use treatment barriers among Latino, White, and Black participants with SUD found that, compared to White and Black individuals, Latinos expressed more pronounced narratives about attitudinal norm barriers tied to cultural factors and stigma, emphasizing the need for culturally informed services beyond Spanish language support (49). Substance use treatment providers and counselors must actively recognize how systemic racism shapes the institutions in which they operate and critically reflect on how their own racial identity may influence power dynamics in care delivery. By integrating ongoing cultural competency training, providers can develop a deeper understanding of the historical and structural inequities affecting racially minoritized communities, ensuring that care is culturally responsive and informed (15, 16).

In general, we found that the overall service model of RCCs varies little across racial/ethnic community settings. For example, we did not find that RCCs serving Black communities functioned drastically different in terms of services provided from RCCs serving Hispanic/Latino communities or RCCs serving other communities. RCCs serving Black, Hispanic/Latino, and other communities appeared to adhere to a model that offered a variety of recovery support services in addition to educational and social services and activities. Future research should incorporate acceptability measures to establish the perceived agreement and satisfaction of the RCC model across diverse racial and ethnic groups. High rates of racially minoritized individuals expect and experience racial discrimination and medical mistrust of traditional systems of substance use treatment leading to delayed and lower engagement in care (50). More research should be done to see if newer substance use treatment models like RCCs—rooted in community-oriented, socioeconomic care, and peer support—may support lower perceived and experienced racial discrimination, increasing acceptability and treatment outcomes (e.g., engagement and completion rates). Indeed, past research has highlighted the ability of PRSS to support low-income ethnic and racially minoritized individuals in their recovery journey in a community resource center (51). PRSS was able to help individuals in this setting who were interested in addressing their substance use by assisting them in overcoming barriers to care and by linking them to substance use treatment. Fifty-two percentage of individuals who were linked to treatment remained in treatment at the 30-day follow-up period (51). Additionally, PRSS has shown effectiveness in engaging individuals in harm-reduction services such as syringe exchange programs, which are important in reducing the potential for overdoses and infections (52) and are notably highlighted as an important service to help address substance use health disparities (12). Given this past research, which speaks to the potential of PRSS, and our findings demonstrating RCC engagement of marginalized communities, the RCC model indicates promise to be an important resource for long-term recovery support service. Additionally, while stigma might hinder marginalized populations from engaging in treatments like medications for SUD, previous studies have shown that RCCs are both welcoming to individuals taking medications and have connections with more formal healthcare settings, such as clinics that provide these medications (29). These findings suggest that RCCs provide promise for helping to overcome racial and ethnic disparities in SUD treatment and long-term recovery.

## Limitations

This secondary data analysis was conducted using data that was collected in 2022. Since then, the number of RCCs in the United States has continued to increase (i.e., in 2022, our team was able to identify 198 RCCs nationwide, 122 of which participated in this survey; we have since then learned of 39 more RCCs, which is likely an underestimate of how many more RCCs have opened their doors since 2022). This means that not all existing RCCs nationwide were captured in the survey (29). Due to this growth, previously non-existent disparities within the RCC framework may have emerged. The significance of any group differences found need to be interpreted with caution: the number of variables we examined was high, which increases the risk of Type I error (false positives), especially given the number of *p*-values reported near the conventional threshold (*p* < 0.05). However, in this first study to examine potential racial/ethnic disparities in the context of RCCs, we aimed to include all possible leads ensuring that all differences identified could be examined for replicability in future studies. At the same time, the dataset was relatively small (*n* = 122), thereby providing limited statistical power to detect group differences, especially for comparisons between RCCs serving Black (*n* = 23) and Hispanic/Latino (*n* = 18) communities. Additionally, this survey assessed characteristics of RCCs through self-reporting from RCC directors or delegates, which may not capture the fullness of the RCC model, footprint, services, or linkages. Future studies should prioritize obtaining more objective or observational data on RCCs as the data collection infrastructure in RCCs develops.

This survey did not assess engagement in cultural activities held at RCCs, which may be an important factor for engaging Black and Hispanic/Latino communities (36). Lastly, the data were limited regarding the provision of harm reduction tools and information. This survey only asked about Narcan (naloxone) training and/or distribution; other services, such as the provision of clean syringes or fentanyl test strips, were not assessed. Future research should examine the ability of RCCs to support harm reduction approaches in a landscape of variably restrictive state legislations and community attitudes, which may illuminate different pathways and opportunities for reducing stigma around harm reduction and reducing harm in the community (53, 54).

## Conclusion

Our results indicate that RCCs successfully engage Black and Hispanic/Latino people with their services and activities. This is an important contribution, given widespread racial/ethnic disparities in accessing and engaging with SUD treatment. These results strengthen the rationale for the current rapidly growing investment in RCCs and the services they offer, providing hope for closing currently existing gaps in providing SUD-related recovery support and care.

## Data Availability

The original contributions presented in the study are included in the article/Supplementary material, further inquiries can be directed to the corresponding author.
