# Supplementary material for: Comparing recovery community centers (RCCs) serving Black, Hispanic/Latino, and other communities: an exploratory secondary data analysis of a nationwide survey of RCC directors
Source: Front Public Health. 2025 Jul 2;13:1532488. doi: 10.3389/fpubh.2025.1532488 (PMC12263450; doi:10.3389/fpubh.2025.1532488)

Supplementary Online Content

1. Supplemental methods.
2. Supplemental Table 1. Levene's tests for homogeneity of variance and Shapiro-Wilk’s tests for normality of error variance on all variables analyzed using ANOVA.
3. Supplemental Table 2. Residual plots for continuous variables from ANOVA models using the original variables vs. using the log-transformed variables.

This supplementary material has been provided by the authors to give readers additional information about their work.

Supplemental methods:

To assess the validity of statistical assumptions for parametric tests for continuous and count variables, we conducted Levene's and Shapiro-Wilk tests to examine equality of variances and normality, respectively. We tested these assumptions for both the original variable as well as log-transformed versions of the variable and obtained Q-Q plots and histograms of the ANOVA residuals. To further examine the impact of potential skewness within our comparison groups, we employed both parametric (ANOVA and ANOVA of log-transformed values) and non-parametric analyses (Wilcoxon rank-sum tests). Based on these assessments, we report (1) p-values from ANOVAs of log-transformed variables for variables with skewed distributions in raw data that improved after log-transformation, including Number of years in operation, Number of paid staff, Number of volunteer staff at your RCC, 60+ years, Other (gender), Black / African American, More than one race, Ethnicity (% Hispanic); and (2) non-parametric results for select variables where assumptions for parametric analysis were not met even after log-transformation, including data for categories with limited sample sizes in Table 1, such as "Other" genders, American Indian or Alaskan Native race, Asian race, and Native Hawaiian or Pacific Islander race, as well as for the variable *Openness of RCC to medication-assisted treatment* (scale 1–5) in Table 4.

Supplemental Table 1. Levene's tests for homogeneity of variance and Shapiro-Wilk's tests for normality of error variance on all variables analyzed using ANOVA.

Variable	Variable transformation	Levene's test	Shapiro-Wilk's test
		p-value	p-value
**RCC Logistics (Table 1)**			
Number of years in operation	Original	0.098	<0.0001
	Log-transformed	0.5819	0.2812
Number of paid staff	Original	0.0067	<0.0001
	Log-transformed	0.7538	0.0378
Number of volunteer staff at your RCC	Original	0.6538	<0.0001
	Log-transformed	0.5042	0.0068
**RCC Footprints (Table 1)**			
Number of RCC members last year	Original	0.1718	<0.0001
	Log-transformed	0.6531	0.0756
Number of active RCC members last month	Original	0.8409	<0.0001
	Log-transformed	0.7776	0.1410
**RCC Demographics (Table 1)**			
Age: <25 years	Original	0.3253	<0.0001
	Log-transformed	0.7639	<0.0001
Age: 25-59 years	Original	0.8763	0.0105
	Log-transformed	0.6187	<0.0001
Age: 60+ years	Original	0.0375	<0.0001
	Log-transformed	0.7617	<0.0001
Gender: % male	Original	0.0603	0.0085
	Log-transformed	0.0066	<0.0001
Gender: % female	Original	0.39	0.0002
	Log-transformed	0.6963	0.015
Gender: % other	Original	0.0495	<0.0001
	Log-transformed	0.3306	<0.0001
Race: % American Indian or Alaskan Native	Original	0.3614	<0.0001
	Log-transformed	0.1693	<0.0001
Race: % Asian	Original	<.0001	<0.0001
	Log-transformed	0.0002	<0.0001
Race: % Black / African American	Original	0.0177	<0.0001
	Log-transformed	0.2452	0.0002
Race: % Native Hawaiian or Pacific Islander	Original	<.0001	<0.0001
	Log-transformed	<.0001	<0.0001

Race: % White	Original	0.0369	0.0009
	Log-transformed	<.0001	<0.0001
Race: % more than one race	Original	0.0135	<0.0001
	Log-transformed	0.1087	<0.0001
Ethnicity: % Hispanic	Original	0.316	<0.0001
	Log-transformed	0.0455	0.1797
**RCC attitudes towards medication assisted recovery across RCCs serving different communities (Table 4)**			
Openness of RCC to medication-assisted treatment (scale 1-5)	Original	0.1862	<0.0001
	Log-transformed	0.0361	<0.0001

Notes: Significant p-values (p<.05) for Levene's test indicate significant departures from homogeneity of variance between groups; significant Shapiro-Wilk's tests indicate significant departures from normality of residual errors.

<table>
<tr><th colspan="2">Residual plots for continuous variables from ANOVA models using the original variables vs. using the log-transformed variables.</th></tr>
<tr><th>Variable</th><th>Residual Plots</th></tr>
<tr><td colspan="2">Number of years in operation</td></tr>
<tr><td>Original</td><td>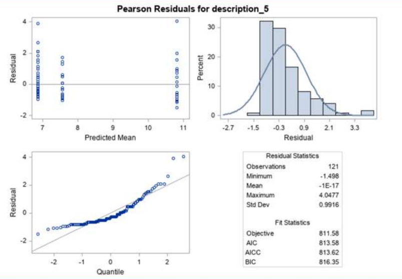
</td></tr>
<tr><td>Log-transformed</td><td>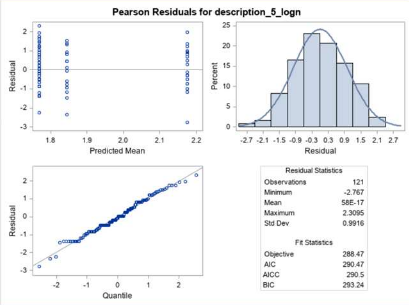
</td></tr>
<tr><td colspan="2">Number of paid staff</td></tr>
<tr><td>Original</td><td>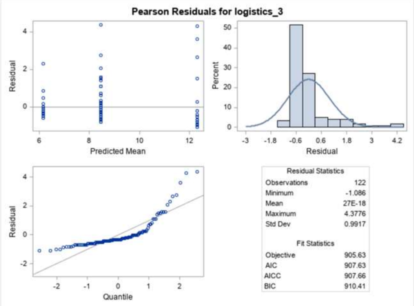
</td></tr>
<tr><td>Log-transformed</td><td>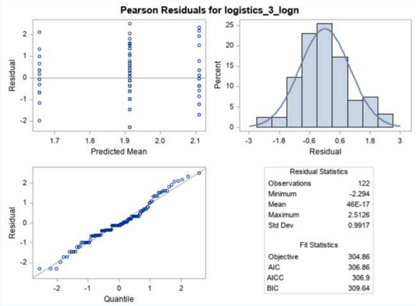
</td></tr>
<tr><td colspan="2">Number of volunteer staff at your RCC:</td></tr>
</table>

Original	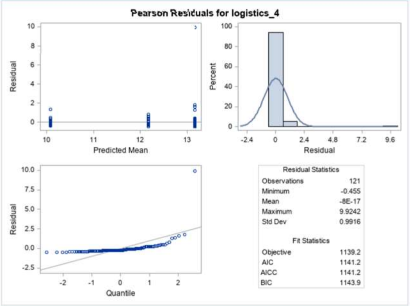
Log-transformed	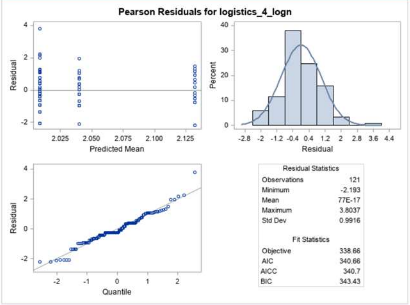
**Number of RCC members last year:**	
Original	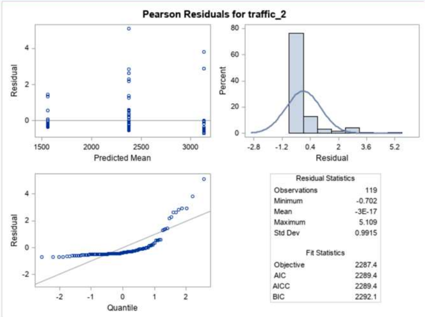
Log-transformed	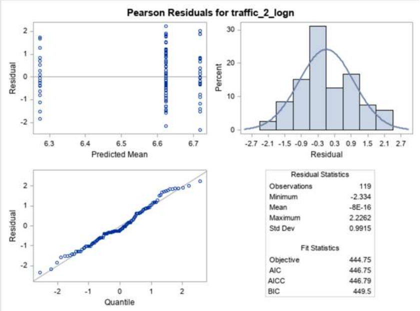
**Number of active RCC members last month**	
Original	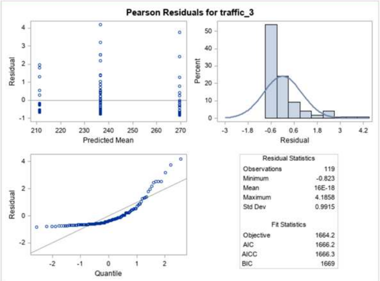

Log-transformed

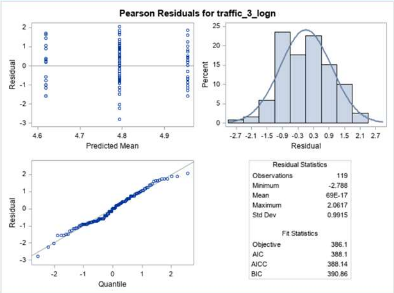

Age

<25 years

Original

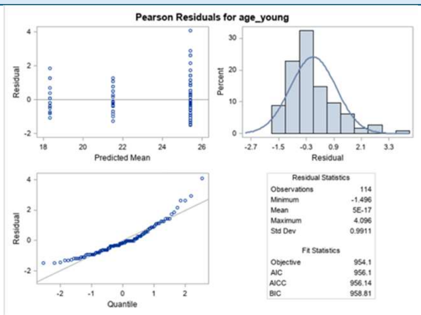

Log-transformed

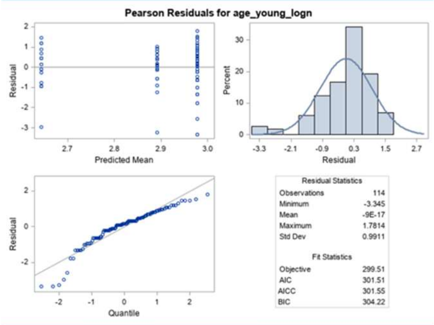

25-59 years

Original

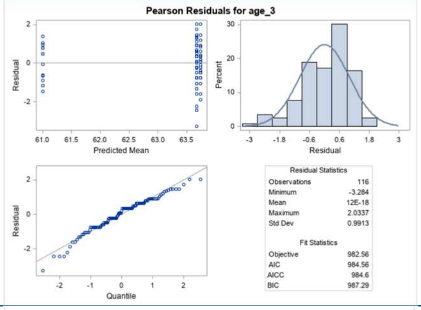

Log-transformed

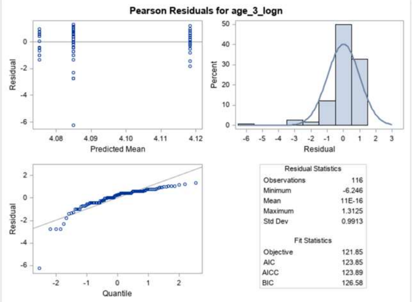

60+ years

Original

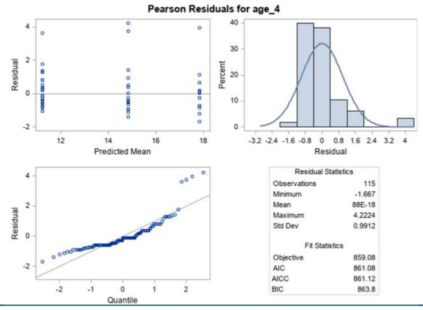

Log-transformed

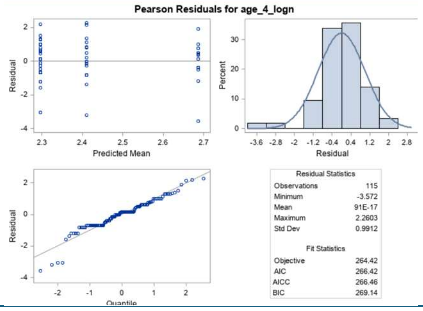

Gender

Male

Original

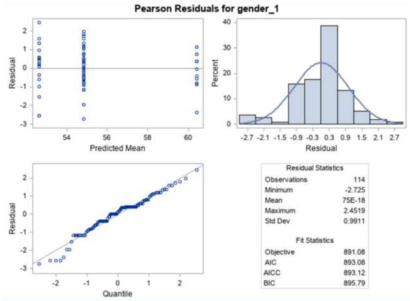

Log-transformed

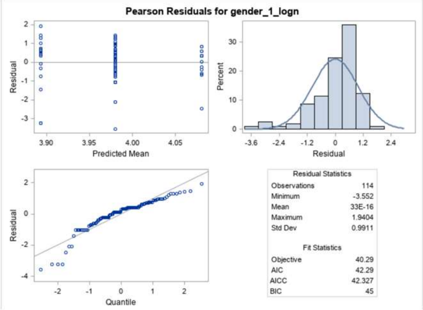

Female

Original	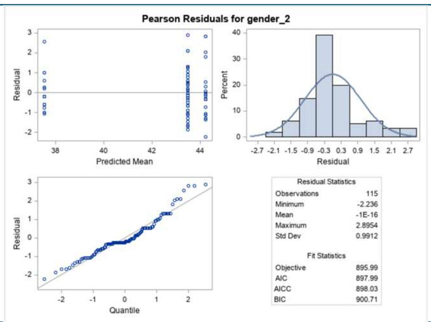
Log-transformed	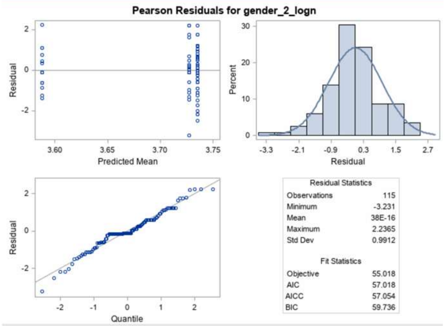
**Other**	
Original	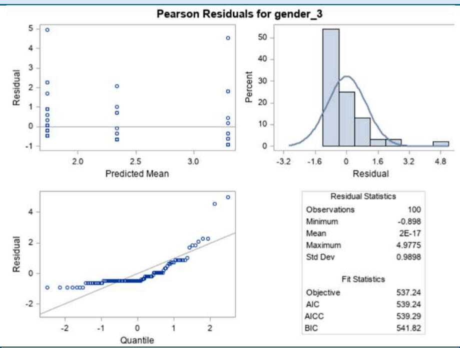
Log-transformed	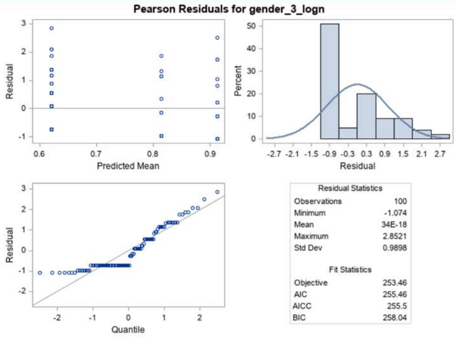
**Race**	
**American Indian or Alaskan Native**	

Original

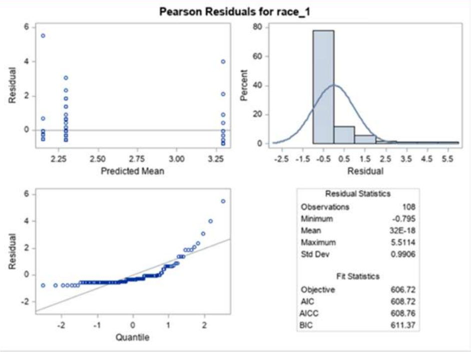

Log-transformed

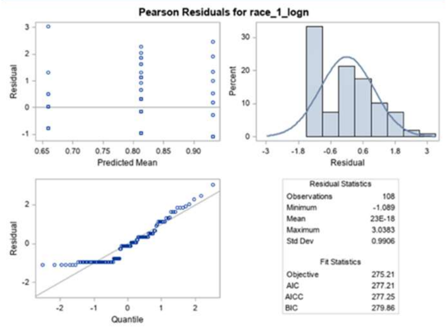

Asian

Original

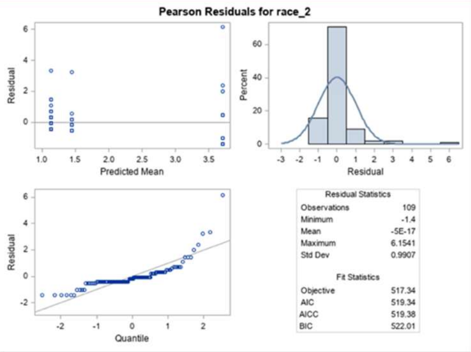

Log-transformed

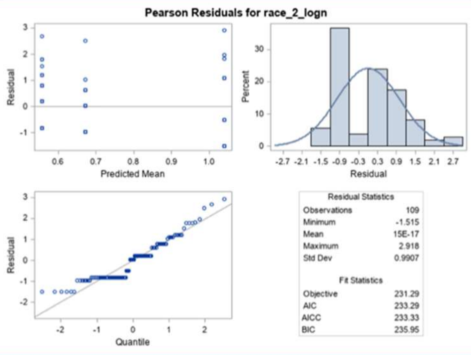

Black / African American

Original

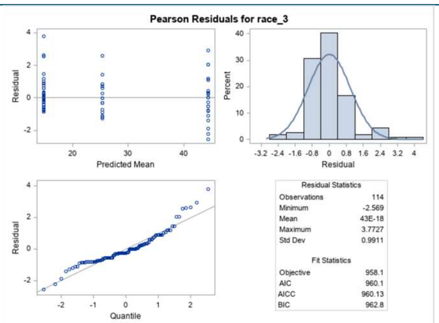

Log-transformed

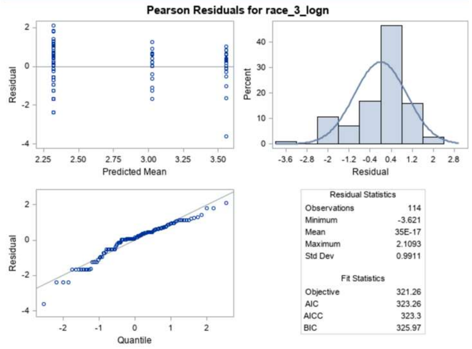

Native Hawaiian or Pacific Islander

Original

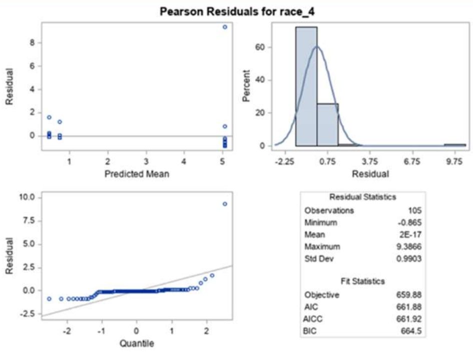

Log-transformed

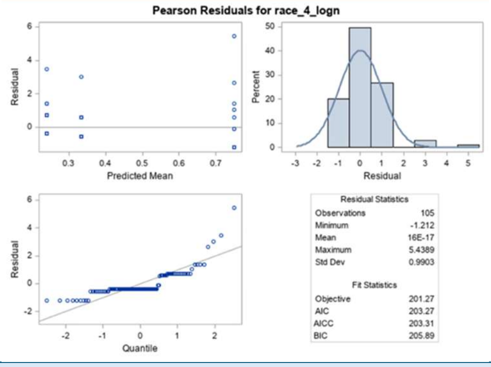

White

<table>
<tr><td>Original</td><td>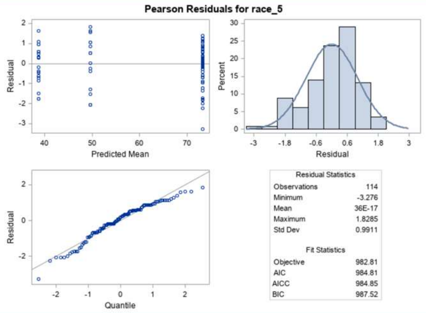
</td></tr>
<tr><td>Log-transformed</td><td>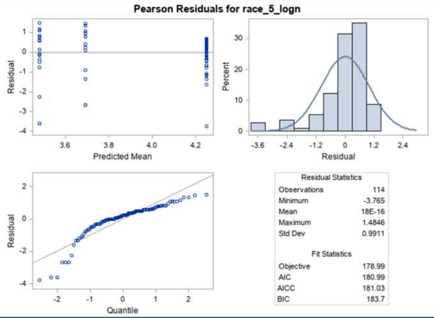
</td></tr>
<tr><td colspan="2">More than one race</td></tr>
<tr><td>Original</td><td>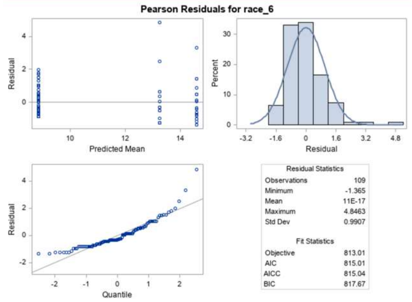
</td></tr>
<tr><td>Log-transformed</td><td>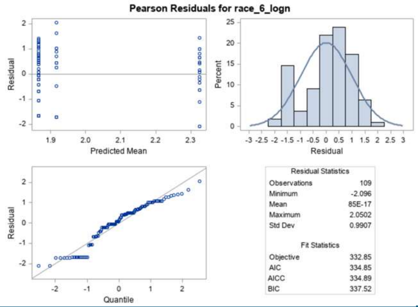
</td></tr>
<tr><td colspan="2"><u>Ethnicity (% Hispanic)</u></td></tr>
<tr><td>Original</td><td>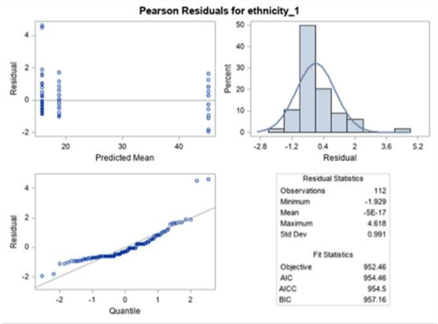
</td></tr>
</table>

Log-transformed

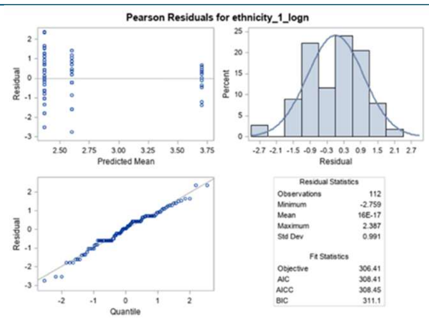

Openness of RCC to medication-assisted treatment, Average score (scale 1-5)

Original

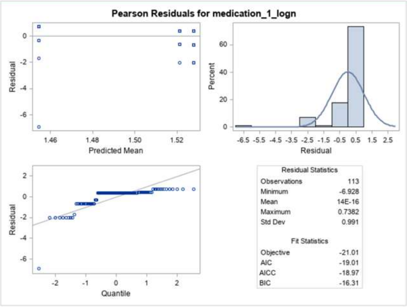

Log-transformed

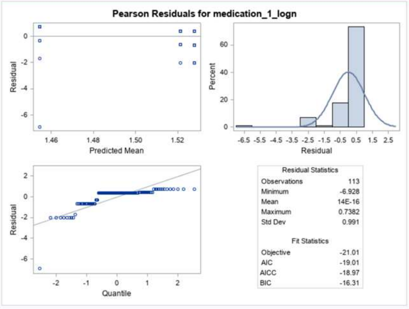

Supplement: Supplementary file 1 [file Data_Sheet_1.pdf]
