# Supplementary material for: Comparing recovery community centers (RCCs) serving Black, Hispanic/Latino, and other communities: an exploratory secondary data analysis of a nationwide survey of RCC directors
Source: Front Public Health. 2025 Jul 2;13:1532488. doi: 10.3389/fpubh.2025.1532488 (PMC12263450; doi:10.3389/fpubh.2025.1532488)
Supplement: Supplementary file 2 [file Table_1.pdf]

Table 0. Comparison of US census data for ZCTAs with RCCs vs nationwide.

US Census code categories	RCCs Director Reported		ZCTA reported		t	p
	mean	(SD)	mean	(SD)		
(for RCCs serving Black communities) % Black	44.4	23.8	51.0	21.0	1.36	0.1881
(for RCCs serving Hispanic/Latino communities) % Hispanic/Latino	45.2	20.5	56.8	16.6	2.02	0.0609

Note. ZCTA = ZIP code tabulation area
